# Polycomb Repressive Complexes: Shaping Pancreatic Beta-Cell Destiny in Development and Metabolic Disease

**DOI:** 10.3389/fcell.2022.868592

**Published:** 2022-05-04

**Authors:** Sneha S. Varghese, Sangeeta Dhawan

**Affiliations:** Department of Translational Research and Cellular Therapeutics, Arthur Riggs Diabetes and Metabolism Research Institute, City of Hope, Duarte, CA, United States

**Keywords:** polycomb, beta cells, differentiation, maturation, proliferation, epigenetics, diabetes

## Abstract

Pancreatic beta-cells secrete the hormone insulin, which is essential for the regulation of systemic glucose homeostasis. Insufficiency of insulin due to loss of functional beta-cells results in diabetes. Epigenetic mechanisms orchestrate the stage-specific transcriptional programs that guide the differentiation, functional maturation, growth, and adaptation of beta-cells in response to growth and metabolic signals throughout life. Primary among these mechanisms is regulation by the Polycomb Repressive Complexes (PRC) that direct gene-expression *via* histone modifications. PRC dependent histone modifications are pliable and provide a degree of epigenetic plasticity to cellular processes. Their modulation dictates the spatio-temporal control of gene-expression patterns underlying beta-cell homeostasis. Emerging evidence shows that dysregulation of PRC-dependent epigenetic control is also a hallmark of beta-cell failure in diabetes. This minireview focuses on the multifaceted contributions of PRC modules in the specification and maintenance of terminally differentiated beta-cell phenotype, as well as beta-cell growth and adaptation. We discuss the interaction of PRC regulation with different signaling pathways and mechanisms that control functional beta-cell mass. We also highlight recent advances in our understanding of the epigenetic regulation of beta-cell homeostasis through the lens of beta-cell pathologies, namely diabetes and insulinomas, and the translational relevance of these findings. Using high-resolution epigenetic profiling and epigenetic engineering, future work is likely to elucidate the PRC regulome in beta-cell adaptation *versus* failure in response to metabolic challenges and identify opportunities for therapeutic interventions.

## Introduction

The pancreatic beta-cells serve as body’s glucose sensors and produce the hormone insulin to maintain systemic glucose homeostasis. Progressive dysfunction and death of beta-cells and the consequent insulin insufficiency is central to the pathogenesis of both type 1- and type 2-diabetes (T1D and T2D) (Ganda et al., 1984; Porte, 1991; Bingley et al., 1992; Weir and Bonner-Weir, 2004; Akirav et al., 2008). Collapse of cellular identity marks beta-cell dysfunction and precedes beta-cell demise (Talchai et al., 2012; Guo et al., 2013; Hunter and Stein, 2017). The limited regenerative capacity of adult beta-cells, which further declines with age, presents an additional challenge in diabetes (Teta et al., 2005; Krishnamurthy et al., 2006; Rankin and Kushner, 2009; Tschen et al., 2009). Understanding the mechanisms that govern beta-cell homeostasis and are disrupted in diabetes is therefore essential for developing therapeutic strategies to replenish the functional beta-cell deficit. Of particular interest are molecular mechanisms that coordinate the transcriptional programs underlying beta-cell health. Epigenetic mechanisms represent an ideal candidate as they direct the spatio-temporal control of gene-expression patterns and cellular phenotypes in response to environmental cues (Bird, 2007; De Jesus and Kulkarni, 2014; Avrahami and Kaestner, 2019). They mediate the context specific interpretation of genetic information and include histone modifications, DNA methylation, chromatin remodeling, and non-coding RNAs (Allis and Jenuwein, 2016). These modules often work in tandem and can serve to either activate or repress transcription (Dhawan and Natarajan, 2019). Polycomb group (PcG) proteins are a family of epigenetic regulators that play a critical role in cellular self-renewal and differentiation and are dysregulated in many developmental disorders and cancers (Imagawa et al., 2017; Blackledge and Klose, 2021a; Blackledge and Klose, 2021b; Luo et al., 2021; Piunti and Shilatifard, 2021; Wang et al., 2021). Here, we discuss the contribution of PcG proteins in beta-cell homeostasis from early development to adult life, including the intersection of PcG-dependent transcriptional control with signaling pathways that regulate functional beta-cell mass. We also highlight recent advances that have shaped our understanding of PcG dysregulation in beta-cell pathologies and their potential translational implications.

## The PcG Complexes: A Brief Overview

PcG proteins repress gene-expression through histone modifications and form large multimeric complexes comprising of core and accessory subunits; these include histone modifying enzymes along with proteins that regulate enzyme activity and target-site recognition. Polycomb Repressive Complexes 1 and 2 (PRC1 and 2) are the most well characterized PcG complexes [reviewed in (Blackledge et al., 2015; Blackledge and Klose, 2021a; Blackledge and Klose, 2021b)]; PRC1 mediates the mono-ubiquitylation of histone H2A at lysine 119 (H2AK119Ub) while PRC2 catalyzes mono-, di-, and tri-methylation of histone H3 at lysine 27 (H3K27me1/2/3). PRC1 contains E3 ubiquitin protein ligase Ring1A or B (Really interesting new gene 1) along with Pcgf1–6 (Polycomb group ring finger 1–6) as core components. The canonical PRC1 complexes include Cbx (Chromobox) and Hph (human polyhomeotic homolog) proteins, while the non-canonical complexes contain Rybp (Ring1 and ying yang 1-binding protein) and Kdm2b (lysine-specific demethylase 2B). PRC2 consist of histone methyltransferase Ezh1 or 2 (Enhancer of zeste homolog), Eed (embryonic ectoderm development), Suz12 (Suppressor of zeste 12), and Rbbp4 or 7 (retinoblastoma-binding protein). The PRC2 accessory subunits include Aebp2 (adipocyte enhancer binding protein 2), Jarid2 (jumonji- and AT-rich interaction domain 2), and Pcl (Polycomb-like). While PRC1 and 2 typically colocalize to their targets and function in a sequential manner with PRC2 binding first, recent data suggest that they can bind and modify target regions independently (Blackledge et al., 2015). Although PRCs typically repress gene-expression, PRC-dependent gene activation has been reported (Blackledge and Klose, 2021a). Regardless of their mode of action, PcG complexes serve as important regulators of gene-expression and tissue homeostasis.

## PcG Regulation in Beta-Cells Homeostasis

### Polycomb Dependent Repression in Early Pancreas Development

PcG proteins play an essential role in orchestrating stage-specific transcriptional programs during pancreas differentiation. The pancreas differentiates from definitive endoderm (DE) lineage, specifically the foregut endoderm (Zorn and Wells, 2009). Pancreatic development begins with the emergence of dorsal and ventral buds harboring multipotent progenitors at embryonic day 9.0 (E9.0). The two buds fuse around E12.5 to form the full organ, followed by the differentiation of pancreatic progenitors into endocrine-, acinar-, and ductal-lineages as the organ continues to grow (Gittes, 2009; Zorn and Wells, 2009; Shih et al., 2013; Bastidas-Ponce et al., 2017; Larsen and Grapin-Botton, 2017). The differentiation of pancreatic progenitors to endocrine cells occurs via an intermediate endocrine progenitor stage marked by Neurogenin3 (Neurog3) expression. By the end of gestation, the pancreas acquires its typical structure with islets scattered throughout the organ (Dhawan et al., 2007; Shih et al., 2013).

**FIGURE 1:**
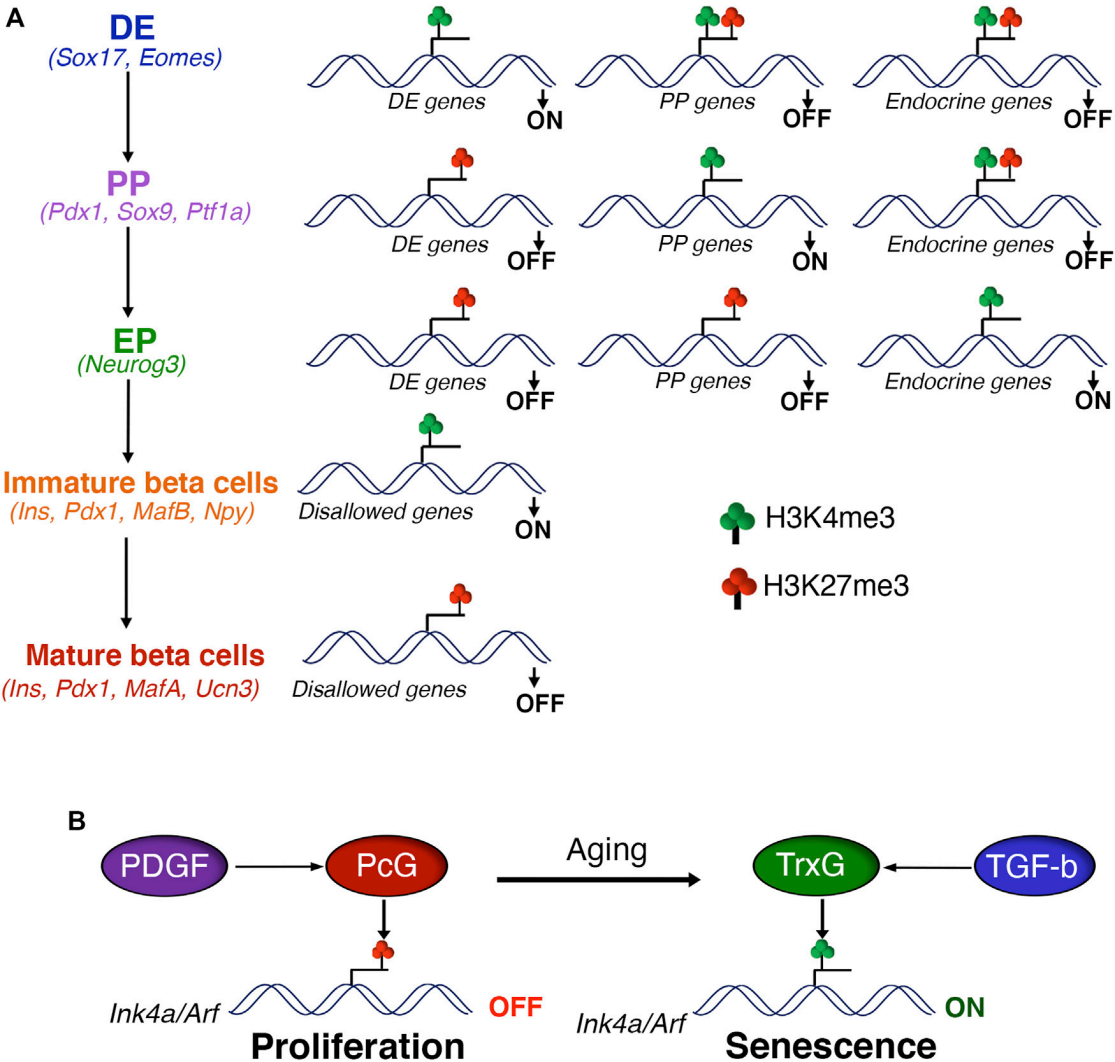
Polycomb control of beta cell differentiation and expansion **(A)** Polycomb proteins regulate the differentiation of pancreatic beta cells *via* sequential H3K27me3 patterning of stage-specific transcriptional programs. Polycomb eviction and consequent removal of H3K27me3 in response to developmental signals is essential for the activation of stage-specific transcription factors during definitive endoderm (DE) specification, marked by the presence of H3K4me3. The transition to pancreatic progenitors (PP) is coupled with the PcG dependent repression of DE genes, marked by gain of H3K27me3 and loss of H3K4me3. DE induction is also hallmarked by the creation of transcriptionally-poised chromatin state at gene promoters of pancreas specification, characterized by the presence of both H3K27me3 and H3K4me3. This regulatory process is reiterated during the subsequent stages of beta cell differentiation, such as PP to endocrine progenitors (EP) and EP to beta cells. Finally, PcG mediated repression of disallowed genes supports the functional maturation of beta cells. Key gene signatures of each developmental stage are noted in parentheses. **(B)** PcG mediated repression of the *Ink4a/Arf* locus is critical for the postnatal beta cell expansion and adaptive response. Age dependent down-regulation of PcG proteins combined with increased TrxG occupancy leads to induction of *Ink4a/Arf* expression and beta cell senescence. Age related changes in the actvitity of growth factor signaling pathways, namely PDGF and TGF-beta, play a key role in the PcG dependent control of beta cell homeostasis.

Studies in mouse and human stem-cell differentiation models have been instrumental in elucidating the role of PcG regulation in early pancreas development, and point to similar regulatory paradigms in both species. The specification of DE is guided by the combinatorial action of Activin/Nodal and Wnt pathways and involves PRC2 eviction and removal of H3K27me3 repressive marks at key endoderm regulatory genes such as SOX17 and EOMES (Xie et al., 2013; Ying et al., 2015). This is mediated by the H3K27me3 demethylase JmjD3, which associates with key stage-specific transcription factors to steer DE lineage specification in response to Activin signaling (Dahle et al., 2010; Jiang et al., 2013; Kartikasari et al., 2013). Loss of H3K27me3 and H2AUb119 from genes such as Eomes primes stem-cells for endoderm specification by DE-inducing signals. Upon induction of DE in human embryonic stem-cells (hESCs), promoters of several lineage-specific factors including pancreas specification genes display loss of the more stable DNA methylation and gain of PcG-dependent H3K27me3 along with the activating histone-H3-lysine 4 trimethylation (H3K4me3) to create transcriptionally-poised chromatin (Gifford et al., 2013), priming the chromatin for future activation by differentiation signals (Figure 1A).

PcG-dependent H3K27me3 patterning also dictates the choice between pancreatic and hepatic lineages during DE refinement, with Ezh2 restricting the pancreatic fate in foregut endoderm (Xu et al., 2011). Mapping of H3K27me3 profiles shows that sequential removal and reinstatement of PcG-dependent repression is a recurrent motif in the rapid induction and silencing of lineage-specific genes throughout lineage progression during pancreatic differentiation. Similar to the loss of PcG-repression at DE promoting genes during DE specification, loss of H3K27me3 guides the induction of pancreatic endoderm (PE) specifying genes during the transition from DE to PE. On the other hand, silencing of transitory genes during lineage progression is achieved by reestablishing PcG-repression in both mice and humans (Xie et al., 2013; Xu et al., 2014). Ezh2 also restricts the induction of pancreatic- and endocrine-progenitor stages of beta-cell differentiation. Accordingly, loss of Ezh2 in pancreatic progenitors in mice enhances endocrine specification (Xu et al., 2014).

Early developmental PcG patterning also shapes the regulatory transcriptional programs relevant to the terminally differentiated beta-cell phenotype. For instance, loss of Ring1b in mouse pancreatic progenitors, but not beta-cells, results in the derepression of target genes in differentiated beta-cells. This suggests that while Ring1b establishes the repression of its targets in pancreatic progenitors, it is maintained via Ring1b-independent mechanisms after beta-cell differentiation (van Arensbergen et al., 2013). Similarly, derepression of a select group of PcG targets during pancreas development allows beta-cells to co-opt a neural transcriptional program, signifying the contribution of early PcG-regulation to differentiated beta-cells. Beta-cells and neurons share a lack of PcG-repression at a select set of genes enriched in transcriptional regulators. Genomic sites harboring PcG-repressed chromatin in pancreatic progenitors overlap with the binding sites for the transcription factor REST (van Arensbergen et al., 2010), which is a suppressor of neuronal genes in nonneuronal cell types and also a potent negative regulator of endocrine differentiation in pancreas (Schoenherr and Anderson, 1995; Rovira et al., 2021). Subsequently, many of these PcG-repressed regions are derepressed during pancreatic endocrine differentiation, concomitant with the loss of REST expression. Notably, loss of REST expression in endocrine differentiation is mediated by PcG-repression (van Arensbergen et al., 2010). Thus, targeted PcG regulation during beta-cell differentiation in conjunction with stage-specific transcriptional regulators guides the establishment of beta-cell specific gene-expression patterns.

### Polycomb Regulation and the Mature Beta-Cell Phenotype

PcG-dependent regulation is critical not just for beta-cell specification but also for their functional maturation. The newly differentiated beta-cells are glucose non-responsive and functionally immature due to a low glucose threshold for insulin secretion (Asplund, 1973; Blum et al., 2012; Dhawan et al., 2015). Beta-cells become glucose-responsive as they undergo a shift in their metabolism in response to changing nutrient quality and growth in neonatal life (Puri et al., 2018). The functionally mature beta-cell phenotype is marked by the restriction of a select set of housekeeping genes termed “disallowed genes”; these include metabolic genes such as the low Km hexokinases (*Hk1 Hk2*)*,* lactate dehydrogenase A (*Ldha*)*,* monocarboxylate transporter-1 (*Mct1/Slc16a1*)*,* and transcription factors such as REST. Repression of these genes is essential for the establishment of glucose sensing and functional maturation (Zhao et al., 2001; Quintens et al., 2008; Thorrez et al., 2011; Schuit et al., 2012; Lemaire et al., 2016; Lemaire et al., 2017).

The restriction of disallowed genes is directed by PcG-dependent H3K27me3 modifications (van Arensbergen et al., 2010; Thorrez et al., 2011). Specifically, Ring1b dependent chromatin modulation in mouse pancreatic progenitors marks disallowed genes for repression in mature beta-cells. Accordingly, ablation of *Ring1b* in pancreatic progenitors leads to impaired beta-cell function without any change in beta-cell mass (van Arensbergen et al., 2013). Similarly, Eed directs the epigenetic silencing in terminally differentiated beta-cells and is essential for maintaining beta-cell identity and function. Beta-cell specific loss of Eed leads to progressive dedifferentiation and dysfunction, resulting in diabetes (Lu et al., 2018). The role of PcG-regulation in beta-cell identity is underscored by the observation that beta-cell hallmark genes involved in transcriptional control are bivalently marked in alpha-cells by H3K27me3 and H3K4me3 (Bramswig et al., 2013), permitting alpha-to-beta cell-fate plasticity which may be useful under conditions of beta-cell deficit.

Prior work has shown that DNA methylation directs the restriction of disallowed genes during beta-cell maturation (Dhawan et al., 2015). PcG proteins and DNA methylation have been shown to coordinate gene regulation in other contexts (Viré et al., 2006; Li et al., 2018; McLaughlin et al., 2019), suggesting that PcG-dependent histone modifications may serve as a framework for the elaboration of DNA methylation patterns to silence disallowed genes. The significance of PcG-regulation in beta-cell maturation is showcased by the striking differences in H3K27me3 profiles between endocrine cells differentiated from hESCs *in vitro,* or *in vivo* after transplantation in a murine host. The *in vivo* differentiated endocrine cells are functionally more mature and display transcriptional and chromatin profiles closely resembling functional human islets. In contrast, the endocrine cells differentiated *in vitro* retain PcG-repression at key endocrine genes, corresponding to their poor functionality (Xie et al., 2013). These data provide important insights into the epigenetic roadmap essential for generating functional beta-cells from hESCs, which has been a challenge in the field.

### Polycomb Control of Beta-Cell Growth and Adaptive Capacity

PcG-regulation also controls the transcriptional programs involved in the postnatal growth and adaptation of beta-cells. Beta-cell replication is a key mechanism underlying postnatal beta-cell mass maintenance, and supports growth, regeneration, and adaptation to metabolic challenges (Georgia and Bhushan, 2004). The burst of beta-cell differentiation in early fetal life is followed by a wave of replication dependent beta-cell expansion during the late fetal to early postnatal growth phase (Finegood et al., 1995; Rane et al., 1999; Georgia and Bhushan, 2004; Kulkarni, 2005; Meier et al., 2008). The rates of replication decline as beta-cells gradually become quiescent and functionally mature in postnatal life, suggesting an inverse correlation between function and proliferation (Puri et al., 2018). Functional mature beta-cells can expand in response to increased beta-cell workload (Stolovich-Rain et al., 2015), and this adaptive capacity declines with age (Teta et al., 2005; Krishnamurthy et al., 2006; Rankin and Kushner, 2009; Tschen et al., 2009).

Genetic loss of function models have uncovered an essential role for PcG regulation in the establishment and maintenance of beta-cell mass in postnatal life. Mice with beta-cell specific deletion of *Ezh2* display reduced beta-cell proliferation and mass, resulting in hyperglycemia and diabetes (Chen et al., 2009). Mice with systemic loss of polycomb protein *Bmi1*/*Pcgf4* display a similar inability to expand beta-cell mass in the neonatal growth phase (Dhawan et al., 2009). Lack of *Ezh2* and *Bmi1* in beta-cells also renders them unable to regenerate in response to injury. These studies further showed that derepression of the *Ink4a/Arf* locus upon PcG ablation is the primary mechanism for loss of beta-cell proliferative capacity (Chen et al., 2009; Dhawan et al., 2009). The *Ink4a/Arf* locus (also known as *Cdkn2a*) encodes cell cycle inhibitors p16^Ink4a^ and p19^Arf^, which accumulate in beta-cells with age and account for the age dependent decline in beta-cell proliferative capacity (Krishnamurthy et al., 2006). Ezh2 plays a dichotomous role in the regulation of beta-cell mass before and after beta-cell specification. Ablation of Ezh2 at different stages of pancreas development in mice demonstrated that while Ezh2 restricts beta-cell differentiation, it also serves to promote beta-cell proliferation in postnatal life by repressing *Ink4a/Arf* (Xu et al., 2014). Notably, the phenotype of beta-cell Ezh2 knockout (KO) is distinct from that of Eed KO, being milder and marked by proliferation defects as opposed to beta-cell dysfunction and severe diabetes in Eed KO (Chen et al., 2009; Lu et al., 2018). Given that loss of Eed disrupts PRC2 complexes containing both Ezh2 and Ezh1 (Xie et al., 2014), the differences between Eed and Ezh2 KO suggest that Ezh2 and Ezh1 complexes play distinct roles in beta-cell homeostasis.

Beta-cells undergo a form of permanent growth arrest with aging, termed replicative senescence, as a result of de-repression of the *Ink4a/Arf* locus in both mice and humans. The levels of Ezh2 decline drastically with aging, corresponding to reduced Ezh2 and Bmi1 recruitment along with loss of H3K27me3 and H2Aub119 marks at the *Ink4a/Arf* locus (Chen et al., 2009; Dhawan et al., 2009). This is accompanied by the recruitment of trithorax G (TrxG) complex, which contains the histone methyltransferase Mll1 and histone demethylase JmjD3 (Dhawan et al., 2009). These two enzymes mark the chromatin with activating histone H3 lysine 4 trimethylation (H3K4 me3) and remove H3K27me3, respectively, and establish transcriptionally active chromatin at the *Ink4a/Arf* locus. In agreement with this, while ectopic expression of Ezh2 in beta-cells can promote beta-cell expansion in young mice, it is unable to reverse the age-dependent accumulation of p16^Ink4a^ in old animals and requires the additional downregulation of TrxG complex to stimulate beta-cell replication (Zhou et al., 2013). PcG-dependent control of beta-cell replication is intimately linked with age-related changes in signaling pathways. Platelet-derived Growth Factor (PDGF) signaling is essential for Ezh2 expression in beta-cells and promotes beta-cell replication. The levels of PDGF receptor (PDGFR) decline with age, resulting in reduced Ezh2 expression and proliferative decline (Chen et al., 2011). On the other hand, Transforming Growth Factor-beta (TGFb) signaling promotes the recruitment of TrxG complex to the *Ink4a/Arf* locus with age and contributes to loss of proliferative capacity and replicative senescence (Dhawan et al., 2016) (Figure 1B). These studies also highlight the conservation of growth-factor dependent control of *Ink4a/Arf* locus in beta-cells between mice and humans (Chen et al., 2011; Dhawan et al., 2016).

Comprehensive epigenetic mapping of murine beta-cells shows that the transcriptional programs promoting proliferation are epigenetically repressed with age while those associated with beta-cell function are potentiated, with underlying age-dependent changes in H3K27me3 profiles (Avrahami et al., 2015). Specifically, distal regulatory elements that lose DNA methylation with age are depleted in H3K27me3 marks, enriched in binding sites for beta-cell transcription factors, and tend to reside near genes related to beta-cell function. On the other hand, proximal promoters appear to gain H3K27me3 marks with aging regardless of their DNA methylation status, a theme also observed in other self-renewing cell types (Avrahami et al., 2015). These data suggest that PcG-dependent chromatin modifications coordinate age-associated changes in beta-cell proliferation and function and reveal an epigenetic basis for their inverse relation.

### Polycomb Regulation: Lessons From Beta-Cell Pathologies

Recent studies show that beta-cell failure in both T1D and T2D is coupled with senescence, an irreversible form of cell cycle arrest induced by chronic cellular-stress (Aguayo-Mazzucato et al., 2019; Thompson et al., 2019a; Thompson et al., 2019b; Aguayo-Mazzucato, 2020; Kumari and Jat, 2021). This form of senescence is distinct from the age-related replicative senescence; it involves not only an *Ink4a/Arf* mediated growth arrest but also DNA damage responses, acquisition of Senescence Associated Secretory Phenotype (SASP) characterized by secretion of inflammatory molecules, and the activation of pro-survival mechanisms (Faget et al., 2019; Kumari and Jat, 2021). Although PcG proteins govern the age-related senescence, their role in pathologic beta-cell senescence in diabetes remains to be established. Emerging evidence in other cell-types shows that PcG dysregulation can directly contribute to SASP (Montes et al., 2021). These observations suggests that cellular-stress could disrupt PcG regulation in beta-cells to drive pathologic senescence. Epigenomic profiling of islets in the context of beta-cell dysfunction in mice and humans has uncovered PRC2-dependent repression as a central mechanism that safeguards the mature beta-cell phenotype. A comparison of islets from mice fed with high fat diet (HFD) or control chow showed that HFD induces a dysregulated chromatin state in islets marked by loss of H3K27me3 repression, corresponding to gene-expression profiles indicative of beta-cell dedifferentiation and dysfunction (Lu et al., 2018). Analysis of pancreatic tissue from subjects with T2D revealed an overall reduction of H3K27me3 in islets compared to non-diabetic subjects, the extent of H3K27me3 reduction corresponding to the extent of disease severity (Lu et al., 2018). Transcriptomic profiling of human islets from non-diabetic and diabetic T2D subjects along with profiling of chromatin states in non-diabetic islets showed that the genes upregulated in T2D islets were found to be marked by H3K27me3 in non-diabetic islets, and included transcription factors typically repressed by PRC2 in non-diabetic islets (Lu et al., 2018). These data show that PRC2 dysregulation contributes to transcriptional changes associated with beta-cell dysfunction in human T2D.

Genomic, epigenomic, and transcriptomic analysis of human insulinomas, beta-cell tumors marked by insulin overproduction and hypoglycemia, underscores the regulatory role of PcG complexes in beta-cell expansion. Majority of human insulinomas harbor concurrent mutations of multiple chromatin modifiers including mutations in PcG (*EZH2*, *YY1*, *RING1*, *BMI1*) and TrxG (*MEN1*, *KDM6A*) genes, and often involve copy number gain for one or more PcG proteins. Furthermore, nearly all insulinomas are marked by the overexpression of *EZH2*. This gain of EZH2 function likely explains the hyperproliferative phenotype and the observed dysregulation of transcriptional programs related to proliferation (Wang et al., 2017). Genes upregulated in insulinomas were found to be highly enriched for H3K27me3 in normal beta-cells, indicating loss of PcG-repression in insulinomas. Comparison of gene-expression signatures of proliferating juvenile human and mouse beta-cells with insulinomas revealed *EZH2* as a key regulator of juvenile beta-cell proliferation along with PcG-target *CDKN1C* (encodes cell-cycle inhibitor p57^Kip2^) (Wang et al., 2017). This suggests that PcG regulation of proliferation is a conserved across multiple beta-cell states, but relies on repression of specific cell-cycle inhibitors (p57^Kip2^ vs. p16 ^Ink4a^) controls distinct phases of beta-cell expansion during neonatal growth and aging.

While these studies establish the contribution of PcG dysregulation to phenotypic changes in beta-cell pathologies, it is not yet clear whether the disruption of PcG function plays a causal role in this context or is a downstream consequence of causal triggers of beta-cell failure such as ER-stress and DNA damage. Nonetheless, the involvement of PcG dysregulation in beta-cell failure makes it an attractive therapeutic target for diabetes. However, adult human beta-cells are extremely reticent to mitogenic cues, making it a challenge to develop approaches focused on beta-cell expansion. Studies on aged mice have provided important clues for promoting human beta-cell replication. Experiments involving Ezh2 overexpression and Pdgfr activation showed that concomitant repression of Mll1 recruitment was required to induce beta-cell proliferation in aged mice (Chen et al., 2011; Zhou et al., 2013). Moreover, loss of Mll1 protects beta-cell Eed KO mice from diabetes (Lu et al., 2018). Accordingly, inhibition of TGF-beta signaling using small molecules can induce beta-cell replication in aged mice as well as in adult human islets by downregulating Mll1 binding to *Ink4a/Arf* locus (Dhawan et al., 2016). Direct pharmacological targeting of PcG proteins using small molecules is a rapidly emerging area in onco-therapeutics (Kreso et al., 2014; Shin and Bayarsaihan, 2017; Su et al., 2019; Shukla et al., 2021). Besides being good potential candidates for beta-cell therapies, such small molecules would also allow us to determine causal roles of PcG proteins in beta-cell pathologies. However, such approaches are not beta-cell specific and can trigger proliferation in other cells. This warrants the development of highly specific therapeutic agents and tissue-specific delivery approaches. Non-coding RNAs are known to coordinate locus-specific PcG control in other cell-types and disease contexts (Spitale et al., 2011; Almeida et al., 2020), and can serve as highly specific therapeutic targets (Leung and Natarajan, 2018). Recent advances in locus-specific epigenetic engineering have made it possible to tailor histone modifications and alter gene-expression (Liao et al., 2017; Pulecio et al., 2017), offering a promising avenue for targeting genes involved in beta-cell function and replication for beta-cell replenishment (Ou et al., 2019).

## Summary and Unanswered Questions

While it is evident from the above discussion that PcG proteins play distinct and stage-specific roles in multiple aspects of beta-cell homeostasis (Figure 1), several questions remain. Given that different PRCs can function independently, it will be important to understand how specific PRCs and PcG-dependent modifications are modulated in response to signals that govern beta-cell mass. These include signals linking the environment with beta-cell phenotype, such as nutrient sensing, circadian clock, mechano-transduction, and cellular-stress (Dhawan and Natarajan, 2019). The adaptive nature of beta-cells suggests epigenetic plasticity, a characteristic of PcG-dependent modifications. How does PcG-regulation evolve in response to acute *versus* chronic metabolic challenges as the beta-cell adapts or fails? Metabolic dysregulation in fetal life predisposes to beta-cell failure in adult life (Bansal and Simmons, 2018; Parveen and Dhawan, 2021). Does embryonic PcG-patterning determine future adaptive potential of beta-cells? Given the role of PcG regulation in beta-cell replication and growth in neonatal life, the reciprocal link between replication and functional maturity (Puri et al., 2018), and the collapse of mature beta-cell phenotype upon PcG dysregulation, it is pertinent to ask if PcG proteins play an active role in beta-cell maturation. And whether PcG transcriptional control contributes to the replicative and functional heterogeneity of beta-cells, which is essential to support adaptation (Pipeleers, 1992; Dorrell et al., 2016; Da Silva Xavier and Rutter, 2020; Benninger and Kravets, 2021). It is also not clear if the cellular stress cues associated with beta-cell failure trigger PcG dysregulation, and whether that actively contributes to beta-cell SASP in diabetes. Lastly, while phenotyping and chromatin occupancy studies in murine KOs, hESC differentiation, and human islets suggest that the PcG regulation of beta-cell homeostasis is conserved between the two species, the direct role of PcG in human pancreas development is far from clear. This gap in knowledge and other emerging evidence warrants examination of the effect of human pathogenic PcG mutations and PcG insufficiency (as opposed to total depletion) on beta-cell development and homeostasis. Elucidation of these aspects and identification of PcG regulatory features unique to human beta-cells will facilitate the discovery of highly specific therapeutic targets.
